# Computational Integral Imaging Reconstruction via Elemental Image Blending without Normalization

**DOI:** 10.3390/s23125468

**Published:** 2023-06-09

**Authors:** Eunsu Lee, Hyunji Cho, Hoon Yoo

**Affiliations:** 1 Department of Computer Science, Sangmyung University, Seoul 110-743, Republic of Korea; 2 Department of Intelligent IOT, Sangmyung University, Seoul 110-743, Republic of Korea

**Keywords:** computational integral imaging reconstruction, integral imaging

## Abstract

This paper presents a novel computational integral imaging reconstruction (CIIR) method using elemental image blending to eliminate the normalization process in CIIR. Normalization is commonly used in CIIR to address uneven overlapping artifacts. By incorporating elemental image blending, we remove the normalization step in CIIR, leading to decreased memory consumption and computational time compared to those of existing techniques. We conducted a theoretical analysis of the impact of elemental image blending on a CIIR method using windowing techniques, and the results showed that the proposed method is superior to the standard CIIR method in terms of image quality. We also performed computer simulations and optical experiments to evaluate the proposed method. The experimental results showed that the proposed method enhances the image quality over that of the standard CIIR method, while also reducing memory usage and processing time.

## 1. Introduction

Integral imaging is a well-known technique for visualizing and recognizing three-dimensional objects. Since Lippmann proposed integral imaging technology in 1908 [1], it has been actively studied in various fields, including 3D image recording, 3D visualization, and 3D object recognition [2,3,4,5,6,7,8,9,10,11,12,13,14,15]. Specifically, computational integral imaging exhibits several advantages over traditional 3D imaging techniques, such as the benefit of a full parallax with white light and continuous viewpoints, without the need to wear eyeglasses. It provides a more immersive and realistic viewing experience in 3D visualization and virtual reality applications.

**Figure 1:**
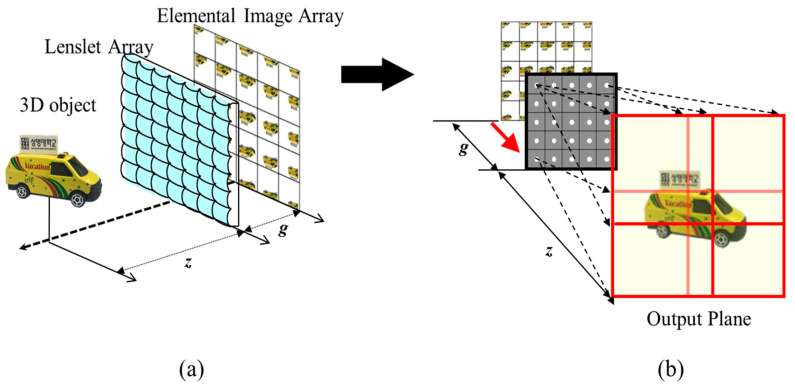
Computational integral imaging system (**a**) pickup and (**b**) computational reconstruction.

The computational integral imaging system is composed of a pickup process and a reconstruction process, as shown in Figure 1. In the pickup process, an image camera captures rays coming from a three-dimensional object and passing through a lenslet array. These recorded rays are known as an elemental image array (EIA). The reconstruction process generates 3D images from the EIA by employing a computational integral imaging reconstruction (CIIR) method. This CIIR method overcomes optical limitations such as lens aberrations and barrel distortion, producing 3D volume images for recognizing 3D objects and estimating their depths.

Generally, computational integral imaging reconstruction (CIIR) methods are based on back projection [16,17,18,19,20,21,22,23,24,25,26,27,28,29,30,31,32,33,34,35,36,37,38,39,40]. The principle of back projection involves projecting 2D elemental images onto a 3D space and overlapping each projected image at a reconstruction image plane [16,17,18,19]. Back projection-based CIIR methods, owing to their straightforward model for ray optics, have been extensively researched for the improvement of 3D imagery [16,17,18,19,20,21,22,23,24,25,26,27,28,29,30,31,32,33,34,35,36,37,38,39,40]. These methods can be categorized into pixel-mapping-based projection, windowing-based projection, and convolution-based projection. Pixel mapping methods project each pixel of an elemental image array into 3D space through a pinhole array [20,21,22,23,24,25], reducing computational costs and improving the visual quality of the reconstructed images. Windowing methods project weighted elemental images into a 3D space, with windowing functions being defined from a signal model of CIIR. The signal model enhances the visual quality of the reconstructed images by eliminating blurring and lens array artifacts [26,27]. Recently, CIIR methods utilizing convolution and the delta function have been introduced to acquire depth information, offering improvements in reconstructed image quality and control over depth resolution [28,29,30,31,32,33,34,35]. In addition, CIIR methods using a tilted elemental image array have been proposed to enhance image quality [36,37]. A depth-controlled computational reconstruction using sub-images or continuously non-uniform shifting pixels was proposed to achieve improved depth resolution and image quality [38,39], and a deep learning-based integral imaging system that uses a pre-trained Mask R-CNN was suggested to avoid blurry areas that are out of focus [40].

Existing methods of computational integral imaging reconstruction (CIIR) typically involve magnification, overlapping, and normalization processes. However, to reduce computational costs, magnification can be replaced with shifting processes. The overlapping process is necessary and cannot be eliminated. The normalization process is also necessary to correct for uneven overlapping artifacts. However, this process demands a significant amount of memory and computing time, equivalent to that of the overlapping process. As the size of each elemental image increases, memory usage and processing time also increase, making this method unsuitable for real-time applications.

In this paper, we present a novel method for computational integral imaging reconstruction (CIIR) using elemental image blending. Our proposed technique eliminates the need for the normalization process to compensate for uneven overlapping artifacts, resulting in decreased memory usage and computational time compared to those of existing methods. We conducted an analysis of the impact of elemental image blending on a CIIR method using windowing techniques. Our model and analysis show that the proposed method is theoretically superior to the standard CIIR method. Additionally, we performed computer simulations and optical experiments to evaluate the proposed method. The experimental results indicate that compared to existing CIIR methods, our method exhibits approximately half the memory usage, along with and improved processing speed. Moreover, the proposed method enhances the image quality over that of the standard CIIR method.

## 2. Conventional Computational Integral Imaging Reconstruction

**Figure 2:**
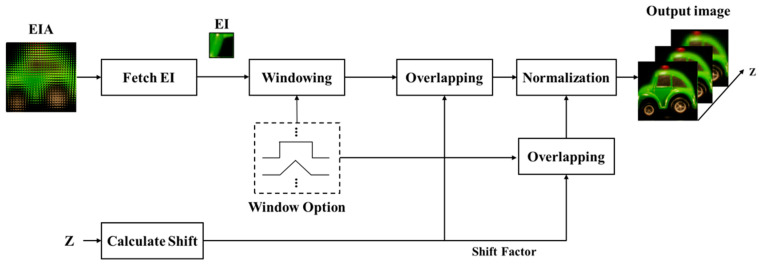
Processes of reconstructing a volume using the standard CIIR method.

The conventional CIIR method is depicted in Figure 2. The model is expanded by the window model to explain existing techniques using the same model. Each elemental image is subjected to a window function, moved according to the shift factor, and then overlapped. The number of overlaps within the reconstructed image plane may be inconsistent, prompting the execution of a normalization process to remove the uneven overlapping artifact. By repeatedly carrying out these operations along the *z*-axis, we can achieve a volumetric reconstruction of a 3D object.

However, the standard CIIR method exhibits some artifacts that lead to a decline in visual quality. During the overlapping process, uneven overlapping artifacts—also known as granular noise—emerge in the reconstructed images [26]. Furthermore, blurring artifacts appear in the out-of-focus areas, and lenslet artifacts arise due to the lenslet’s shape. As the lenslet shape is rectangular, the reconstructed images appear blocky. The window-based CIIR method has been proposed as a means to enhance the quality of reconstructed images by eliminating these artifacts [26,27]. This CIIR approach incorporates a window function in the overlapping process, using a signal model.

The standard CIIR method can be viewed as a CIIR approach that employs a rectangular window. In this case, the rectangular window can be substituted with a different window, such as a triangular or cubic one. Switching the rectangular window to a triangular window reduces blurring artifacts and lenslet artifacts, enhancing the image quality of the reconstructed image [27]. The primary cause of these artifacts is the discontinuity in the windows. Thus, to minimize artifacts, a smooth and continuous window should be implemented to eliminate discontinuity and produce seamlessly reconstructed signals.

**Figure 3:**
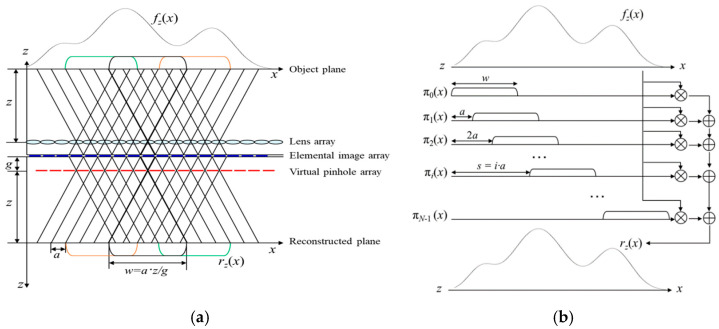
(**a**) 1D optical model of integral imaging and (**b**) its window signal model.

To explain the signal model of integral imaging, we introduce our previously proposed signal model [26]. Figure 3a is an optical model using a 1D pinhole array of an integral imaging system. Figure 3b is a model that abstracts the integral imaging system by introducing a rectangular window function. Here, *f_z_*(*x*) is the intensity signal of an object located at the distance z from the lens array, and *r_z_*(*x*) is the reconstructed signal at the distance z obtained by the back-projecting and overlapping of the picked-up EIA passing through the virtual pinhole array. The EIA pickup process is described as a procedure that windows, inverts, and downscales the *f_z_*(*x*) signal. On the contrary, the reconstruction process is explained as a process that re-inverts, upscales, and overlaps these elemental images. This means that the inversion and scaling effects disappear, leaving only the windowing and overlapping effects. It thus allows for the reconstruction model to be described as a simplified model in Figure 3b.

Based on the model in Figure 3b, the relationship between the original signal *f_z_*(*x*) and its reconstructed signal *r_z_*(*x*) is written as

(1) $$r_{z}\left(x\right)=f_{z}\left(x\right)\sum_{i=0}^{N-1}\pi_{i}\left(\frac{x}{w}\right)=f_{z}\left(x\right)S_{\pi }\left(x\right),$$

where *π_i_*(*x/w*) *= π*_0_((*x-i*⍺)/*w*), ⍺ represents the size of each elemental image, *N* represents the number of elemental images, and *w* represents the size of the window *π*_0_(*x*). Here, the shifted window function (SWF) *π_i_*(*x*) is a shifted version of the window function *π*_0_(*x*), where the function *π*_0_(*x*) = 1, for 0 ≤ *x* ≤ 1, and zero otherwise. In SWF, the shifting factor *s* is a multiple of the elemental signal length ⍺; that is, *s* = *i*·⍺. The sum of the shifted windows is *S_π_*(*x*), and from Equation (1), the original signal *f_z_*(*x*) is obtained as *f_z_*(*x*) = *r_z_*(*x*)/*S_π_*(*x*). Here, the normalization process is considered by the division of a reconstructed image by the summation of SWFs in the window-based CIIR method.

**Figure 4:**
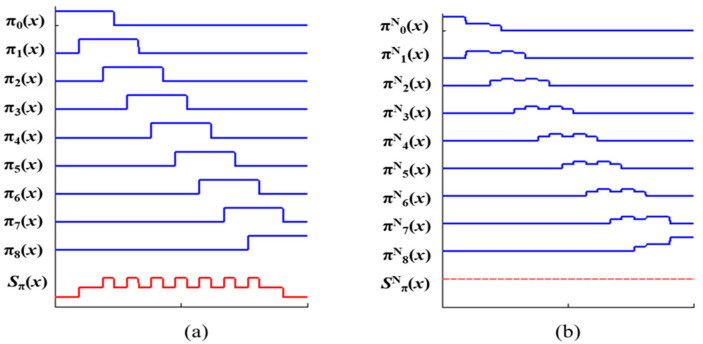
(**a**) Illustration of the sum of nine SWFs in standard CIIR; (**b**) the results of normalization.

**Figure 5:**
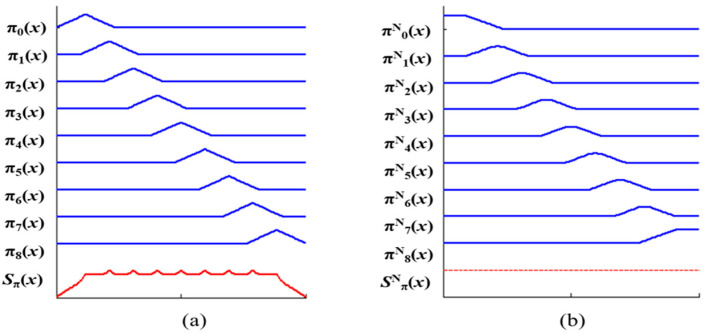
(**a**) Illustration of the sum of nine SWFs in triangular CIIR; (**b**) the results of normalization.

For example, Figure 4a shows nine shifted window functions (SWFs) using a rectangular window function, while Figure 5a displays nine SWFs derived from a triangular window function. The sum of these SWFs, represented as *S_π_*(*x*), is highlighted in red at the bottom of each figure. The function *π^N^_i_*(*x*) in Figure 4b represents the normalized window function of *π_i_*(*x*), achieved by dividing each rectangular window function *π_i_*(*x*) by *S*_π_(*x*); hence, *π^N^_i_*(*x*) = *π_i_*(*x*)/*S*_π_(*x*). As seen in the lower section of Figure 4b, the sum of these normalized SWFs equals one. Figure 5b echoes this concept, presenting normalized SWFs for the triangular window function. It can be observed that the window functions in Figure 5a are simply translated versions of *π_i_*(*x*); however, their normalized window functions can be different due to the shape of the function *S*_π_(*x*), as illustrated in Figure 5b. The differences are particularly noticeable in *π^N^*_0_(*x*) and *π^N^*_8_(*x*). In conclusion, applying normalized window functions to the EIA eliminates the need for the normalization process of the CIIR. Therefore, the development of a method using normalized window functions is important.

## 3. Proposed CIIR Model via Elemental Image Blending

A computational reconstruction method for integral imaging systems is proposed by using elemental image blending. Conventional CIIR methods require a compensation process to normalize the reconstructed images due to uneven overlapping. The normalization process is performed by dividing the reconstructed images by the summation of the SWFs. This process requires additional memory because it needs to record the overlapping numbers for all pixels of the reconstructed images. To eliminate the normalization process, we introduce elemental image blending. Here, the overlapping process is modified with elemental image blending; thus, the normalization process can be canceled out. It turns out that our method saves approximately half of the memory, consequently improving the computation speed. In this section, we describe the proposed method and provide a mathematical analysis of the impact of the elemental image blending technique on CIIR using the window signal model.

**Figure 6:**
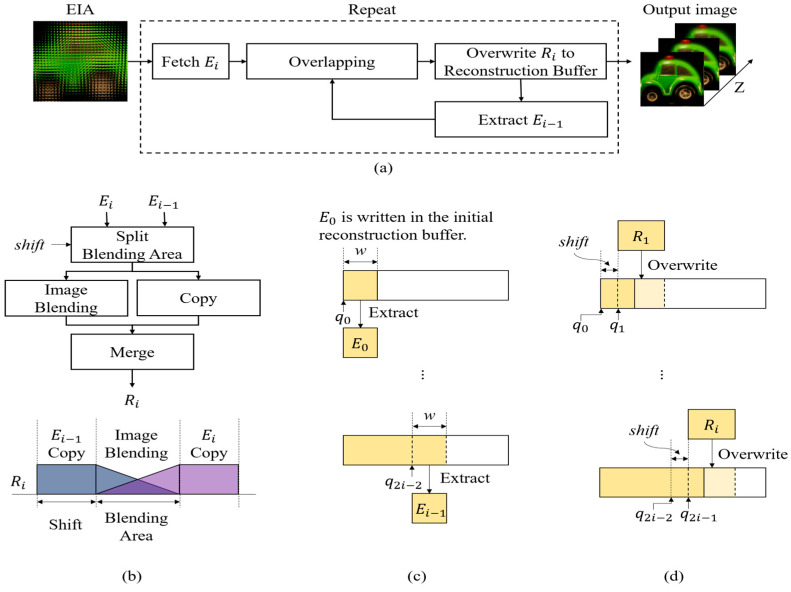
Diagram of the proposed CIIR method based on image blending: (**a**) represents the flowchart of the proposed method, and (**b**) describes the overlapping process; (**c**,**d**) illustrate the $E_{i-1}$ extraction process and the $R_{i}$ overwriting process, respectively.

Figure 6 depicts the proposed CIIR method based on elemental image blending. The flow of the proposed method is as follows: First, two elemental images, $E_{i-1}$ and $E_{i}$, are obtained, and then overlapping is performed using elemental image blending. The resulting image is then overwritten onto the reconstruction buffer. This process is repeated for all the elemental images of the EIA. In the proposed method, the reconstruction buffer is a temporal memory that stores the overlapped image $R_{i}$ of the two elemental images, $E_{i-1}$ and $E_{i}$. The elemental image $E_{i-1}$ is extracted from the reconstruction buffer, while $E_{i}$ is obtained from $i^{th}$ elemental image in EIA. As depicted in Figure 6a, the proposed method consists of the overlapping process using image blending, the extraction process of the elemental image $E_{i-1}$, and the overwriting process of $R_{i}$, as explained in the following paragraphs.

Figure 6b describes the process of overlapping two images, $E_{i-1}$ and $E_{i}$, using image blending. In the overlapping process, there are areas where the two input images overlap and areas where they do not. The area where two images overlap, with blending, is referred to as the blending area. Let the parameter w be the width of the input images, and the parameter a be the shift factor. The overlapping range in $E_{i-1}$ is from a to w, while the overlapping range in $E_{i}$ is from 0 to w−a. Thus, the blending area and the non-blending area are separable. The blending area is blended by the well-known alpha-blending for elemental image blending. We choose the alpha-blending as the elemental image blending due to its simplicity and effectiveness. The blended image is stored in $R_{i}$ from a to w. The non-blending areas of $E_{i-1}$ and $E_{i}$ are copied in $R_{i}$ from 0 to w−a and in $R_{i}$ from w to w+a, respectively. Thus, the images of each area are merged to output $R_{i}.$Figure 6c illustrates the process of extracting $E_{i-1}$ from the reconstruction buffer. The overlapping results are stored in the reconstruction buffer, and thus the extracted image of size w×w from this buffer is different from the elemental image. In the initial reconstruction buffer, the elemental image located at the leftmost position in EIA is written as $E_{0}.$ The initial queue pointer, $q_{0}$, points to the 0th column of the reconstruction buffer. The image of size w×w, extracted from $q_{0}$, is defined as $E_{0}$. As the overlapping and overwriting process repeats, the queue point is updated. $E_{i-1}$ can be obtained by extracting an image of w×w in size from $q_{2i-2}$.

Figure 6d illustrates the process of overwriting $R_{i}$ in the reconstruction buffer. $R_{1}$, the overlapping image of $E_{0}$ and $E_{1}$, is overwritten onto the buffer, starting from $q_{1}$. Here $q_{1}$ is the position obtained by moving $q_{0}$ by a. As the overlapping and overwriting processes are repeated, the queue pointer is updated as

(2) $$q_{i}=q_{i-1}+a.$$

When $R_{i}$ is input in the overwriting process, the queue pointer is updated, and $R_{i}$ is overwritten onto the buffer starting from $q_{2i-1}$.

**Figure 7:**
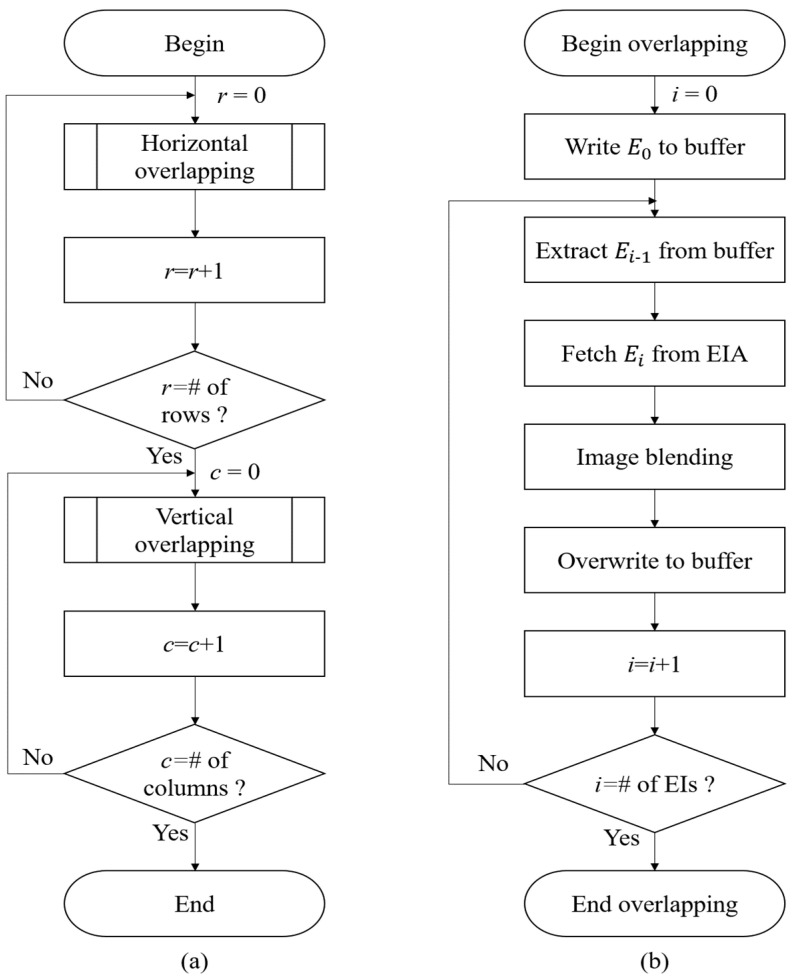
(**a**) Flowchart of the proposed method. (**b**) Horizontal overlapping for each row of EIA.

We also provide the flowchart of the proposed method, as shown in Figure 7. The proposed method employs horizontal overlapping for each row of the EIA, as shown in Figure 7a. Vertical overlapping is applied to the resulting images after horizontal overlapping, which is achieved by the use of horizontal overlapping of the resulting transposed images. Figure 7b depicts the flowchart of the overlapping process. The first elemental image, $E_{0}$, is written in the reconstruction buffer. Subsequently, $E_{i-1}$ is extracted from the reconstruction buffer, and $E_{i}$ is fetched from the EIA for image blending. This process is repeated until the final elemental image is blended in the image blending step.

The proposed method of the overlapping and overwriting processes, as described above, can eliminate the normalization process. Here, we mathematically explain how the normalization process can be eliminated and how our elemental image blending affects the image quality of the reconstructed image.

To analyze the overall CIIR model using elemental image blending, elemental image blending is represented by windowing an elemental image. Here, alpha-blending was used as an example. When two elemental image signals are alpha-blended, two weighted signals, $A_{1}(x)$ and $A_{2}(x)$, are represented as two windowing functions for two elemental images, which are written as

(3) $$A_{1}\left(x\right)\left\{\begin{array}{c} 1,\;(0\leq x<a)\\ \frac{w-x}{w-a},\;(a\leq x<w) \end{array}\right.,\;A_{2}\left(x\right)\left\{\begin{array}{c} \frac{x}{w-a},\;(0\leq x<w-a)\\ 1,\;(w-a\leq x<w) \end{array}\right.$$

**Figure 8:**
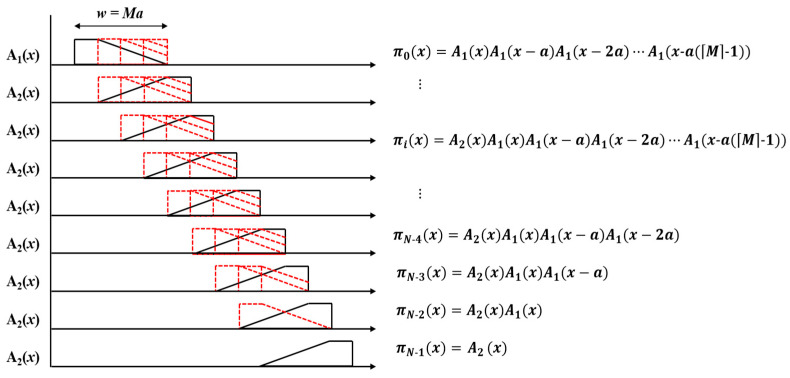
Weight of alpha blending by overlapping, using a window.

As the elemental images overlap, the weights of the signals change due to overlapping, as shown in Figure 8. Accordingly, the weights after overlapping can be represented in different windowing functions. Let the weight of the first elemental image signal be $\pi_{0}(x)$. It is then defined as the product of the shifted function of $A_{1}(x)$,

(4) $$\pi_{0}\left(x\right)=A_{1}\left(x\right)A_{1}\left(x-a\right)A_{1}\left(x-2a\right)\cdots A_{1}\left(x-a\left(\left\lceil M\right\rceil -1\right)\right).$$

Similarly, the weight of the $i^{th}$ signal, $\pi_{i}(x)$, is defined as

(5) $$\pi_{i}\left(x\right)=A_{2}\left(x\right)A_{1}\left(x\right)A_{1}\left(x-a\right)A_{1}\left(x-2a\right)\cdots A_{1}\left(x-a\left(\left\lceil M\right\rceil -1\right)\right),$$

which is the product of $A_{2}(x)$ and the shifted function of $A_{1}\left(x\right),$ when i is between 2 and $N-\left\lceil M\right\rceil -1$. As shown in Figure 8, the $A_{1}(x)$ signal is required for image blending with the next elemental image; thus, its overlap decreases as i increases from $N-\left\lceil M\right\rceil$ to the end.

**Figure 9:**
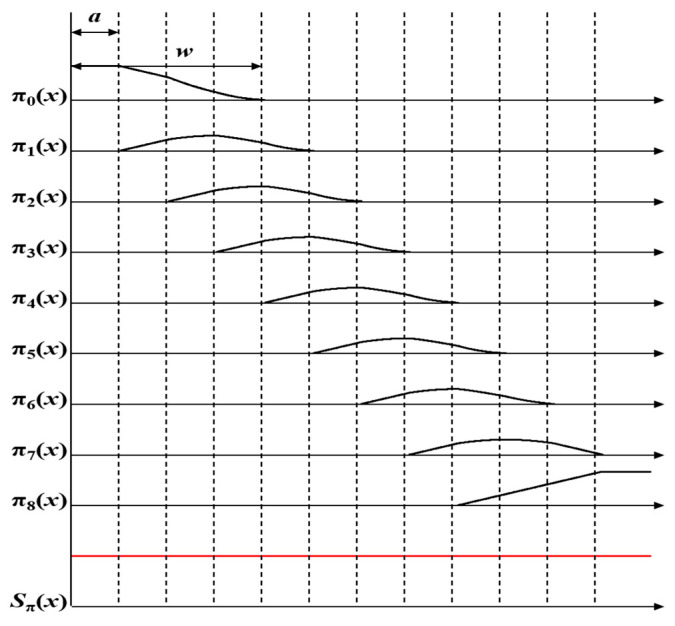
Alpha-blended signal in a shifted window function format.

According to the formulas, the alpha blending signal for each elemental image can be represented in shifted window functions, as shown in Figure 9. Since the summation of window functions is a unit, the original signal can be reconstructed without compensation processes, such as normalization. Note that the form of each window in Figure 9 is a continuous and smooth window. According to the window theory mentioned in Section 2, a continuous window is excellent for removing lenslet artifacts, and it reduces blurring artifacts that occur in the standard CIIR method, which utilizes the rectangular window. Therefore, the proposed method using elemental image blending provides better image quality than does the standard CIIR, while also and requiring less memory and computing time.

## 4. Experiment Results and Discussion

**Figure 10:**
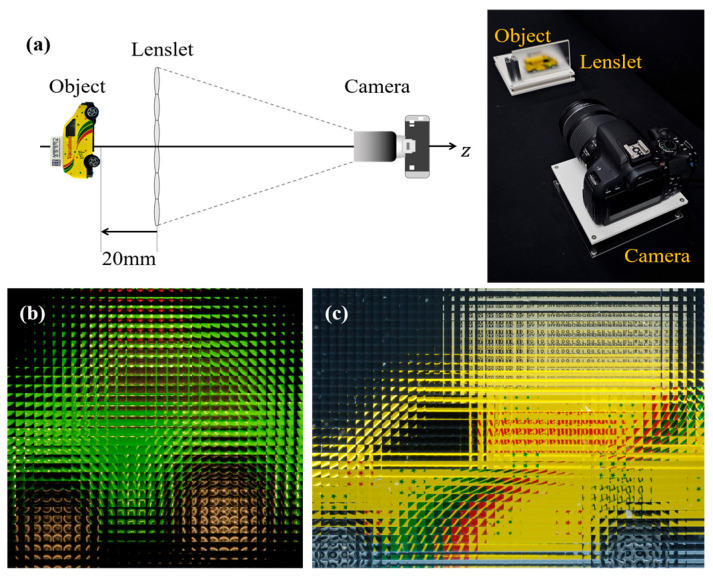
Optical experimental setup and acquired EIAs. (**a**) optical setup (**b**) EIA of the green car (**c**) EIA of the yellow car.

To demonstrate the usefulness of our proposed method, we conducted optical and computational experiments. Figure 10 illustrates the optical experimental setup consisting of lenslets and a CCD camera, along with the acquired EIAs in that environment. The size and focal length of each lenslet is 1.08 mm and 5.2 mm, respectively. The CCD camera in use is the Canon EOS 800D model, equipped with an APS-C size 1.6× crop sensor and an effective pixel count of 24.2 million pixels. Two 3D objects, green and yellow cars, were used in the experiment. The EIA for the green car comprises 32 × 32 elemental images, with each elemental image consisting of 32 × 32 pixels. The EIA for the yellow car consists of 45 × 34 elemental images, and each elemental image has a resolution of 58 × 58 pixels. The resolution of the elemental images can be controlled by adjusting the distance between the camera and the lens array. The location of the 3D objects, $z_{0}$, is around 20 mm. We reconstructed the 3D objects using existing CIIRs with rectangular and triangular windows and our proposed CIIR.

**Figure 11:**
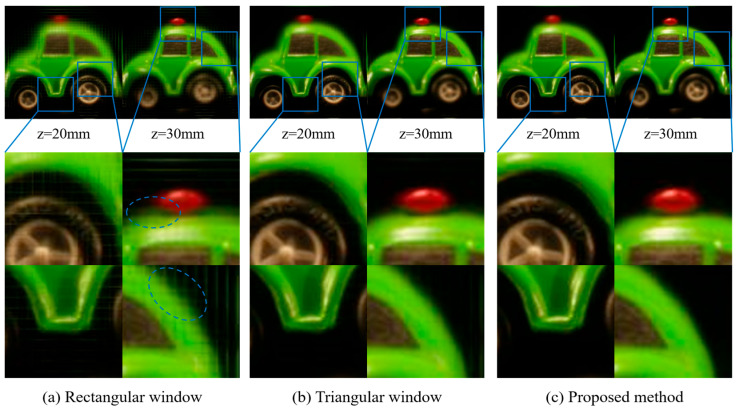
Reconstructed images of the green car using (**a**) a rectangular window, (**b**) a triangular window, and (**c**) proposed method at *z* = *z*_0_ (20 mm) and *z* = *z*_0_ + 10 mm.

Figure 11 shows the reconstructed images of the green car with the output plane locations set at 20 mm and 30 mm. The images in Figure 11a are reconstructed by the standard CIIR with a rectangular window. In the reconstructed image at 20 mm, there is a blocky area due to lenslet artifacts. Similarly, the reconstructed image at 30 mm also shows blocky areas, and the object boundary is defocused and blurry, as highlighted by the dotted ellipses areas. The images in Figure 11c are reconstructed by the proposed CIIR. In the reconstructed image at 20 mm, there are no blocky areas, and the object boundary is clean, compared with those of the standard method. The reconstructed image at 30 mm also shows no blocky areas, and the object boundary is relatively sharp. The images in Figure 11b are reconstructed by the window-based CIIR with a triangular window. The images suffer from less lenslet and blurring artifacts, compared to those obtained by using the CIIR with a rectangular window. Also, they show similar quality to those of the reconstructed images using the proposed CIIR.

**Figure 12:**
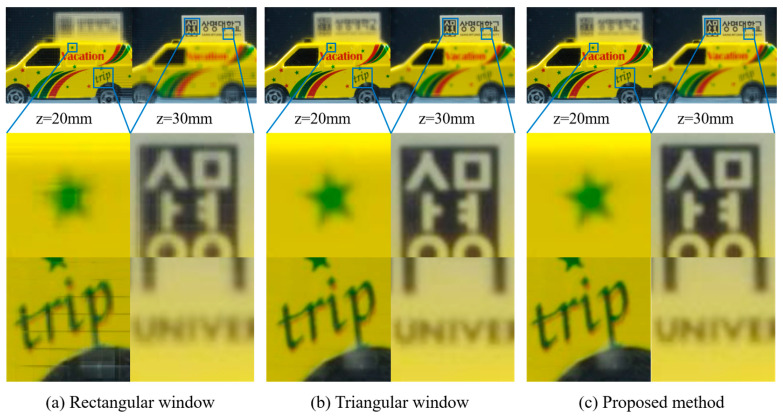
Reconstructed images of the yellow car using (**a**) a rectangular window, (**b**) a triangular window, and (**c**) proposed method at *z* = *z*_0_ (20 mm) and *z* = *z*_0_ + 10 mm.

Figure 12 shows the reconstructed images of the yellow car with the same locations of the output plane as those in the previous experiment. Figure 12a shows the images reconstructed by the window-based CIIR with a rectangular window. As can be seen from the enlarged images, the star and text are blurry due to blurring artifacts, and there are blocky areas due to the lenslet artifacts. On the other hand, the image reconstructed by the proposed CIIR using alpha blending, indicated in Figure 12c, shows reduced blurring artifacts and significantly fewer lenslet artifacts. The image quality of the proposed method is similar to that of the image reconstructed by the window-based CIIR with a triangular window, as shown in Figure 12b.

**Figure 13:**
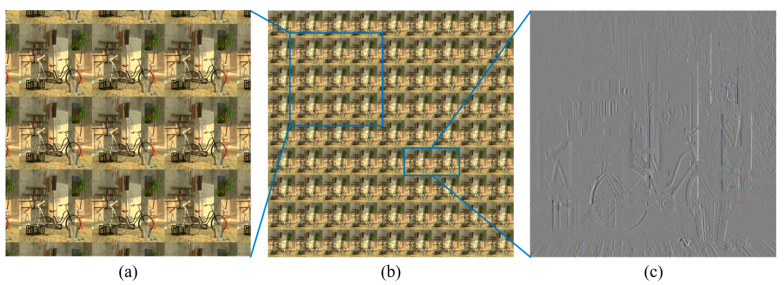
Public light field data from the Heidelberg Collaboratory for Image Processing (HCI) used in the experiment. (**a**,**b**) EIA and its zoomed area of 9 × 9 elemental images from the 81 HCI image files; (**c**) a difference view of two neighboring elemental images.

We conducted another experiment using the public light field dataset provided by the Heidelberg Collaboratory for Image Processing (HCI) [41]. Figure 13a,b displays one of the HCI datasets used in our experiment, called ‘bicycle’. As the HCI data are provided in a set of 81 files, we concatenate these into a 2D array to form an EIA suitable for CIIR. Consequently, the newly prepared EIA comprises 9 × 9 elemental images, each of which has a size of 512 × 512 pixels. Figure 13a presents a magnified section of this EIA.

Note that the characteristics of the HCI data contrast, to some extent, with those of an optical EIA. In general, an EIA directly picked up through a planar lens array exhibits a positive disparity for near objects. The disparity approaches zero as the distance to the objects increases. On the other hand, the disparity of the two nearest elemental images from the HCI data shows positive values for near objects and negative values for distant examples. Moreover, the disparity values present in these HCI images do not exceed 1. These observations can be easily inferred from the elemental image difference, as illustrated in Figure 13c.

To apply the HCI images to CIIR, an EIA from these HCI images should be prepared. Given that the disparity value is less than one, each elemental image is magnified by image interpolation. For addressing negative disparity values, each elemental image is inverted by image flipping. Subsequently, these elemental images are concatenated into a 2D array to construct the EIA. Once prepared, this EIA could be applied to the CIIR methods, including our proposed method, allowing us to evaluate the experimental results.

**Figure 14:**
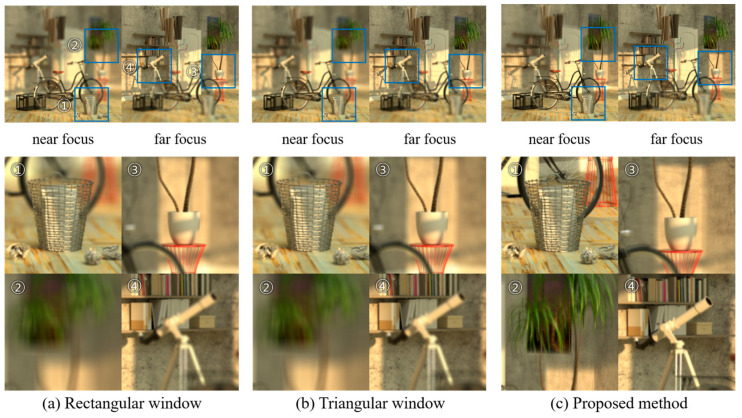
Reconstructed images of ‘bicycle’ data using (**a**) a rectangular window, (**b**) a triangular window, and (**c**) the proposed method.

Figure 14 illustrates the reconstructed images of the ‘bicycle’ dataset, focusing on near and far objects, respectively. For near focus, the shift factor for CIIR is set to 1 pixel. As performed for the distant focus, elemental images were similarly flipped and magnified twice, and they were then applied to the CIIRs, with a shift factor of 1 pixel. Figure 14a,b depicts the images reconstructed from the CIIR methods, based on rectangular and triangular window functions, respectively. Figure 14c shows the images from the proposed method. The areas marked as ① and ③ in Figure 14 represent the images of objects located at a depth in focus. All three methods show clear objects. Areas marked as ② and ④ in Figure 14 represent the images of objects positioned at a depth out of focus.

As depicted in Figure 14c, the images reconstructed from the experiment utilizing the HCI data also show that our method yields relatively sharper images compared to those captured using conventional methods. Notably, this experimental result indicates that our approach significantly broadens the depth of focus relative to the existing methods. Furthermore, the blurring phenomenon in the areas out of focus is reduced in the proposed method, yielding smoother image quality compared to that of the conventional methods.

We repeated a time–memory measurement experiment to demonstrate the usefulness of the proposed method in terms of memory and time efficiency. The experiment was performed by implementing CIIRs with elemental image blending, a rectangular window, a triangular window, and a cubic window in MATLAB R2022a. The experiment used an EIA of 10 × 10 elemental images, with 512 × 512 pixels as the size of each elemental image. The computer used for time measurement was equipped with an Intel^®^ Core™ i7-10700KF CPU @ 3.80 GHz processor and 64 GB RAM.

**Figure 15:**
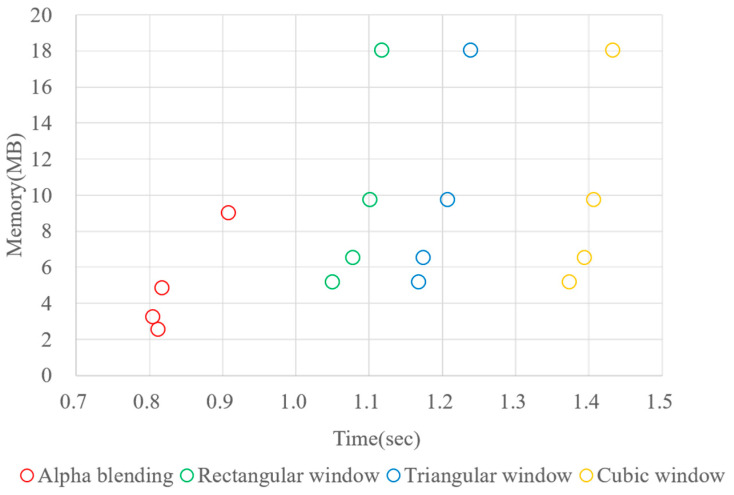
Time–memory scatterplot.

In this experiment, we conducted CIIRs without magnification in order to minimize memory usage. The window-based CIIR methods require memory for normalization, in which the amount of required memory is the same size as the reconstructed image. In contrast, the proposed CIIR requires only the memory for a reconstructed image. The experiment employed a fixed size for both the elemental image and the number of elemental images. For each CIIR method, the shifting factors were varied with values of 8, 16, 32, and 64. Figure 15 shows the simulation results as a scatterplot. Typically, memory requirements increase as the shifting parameter increases in CIIR. Moreover, the computational time required follows the order of the rectangular, triangular, and cubic windows, in accordance with the complexity of CIIR. As shown in Figure 15, the proposed CIIR using alpha blending requires the least time and memory, when compared to the other methods.

A notable point to compare is that the proposed method requires less time and memory than the method using a triangular window, which provides a similar image quality to that of the proposed method in the optical experiment conducted above. The proposed method requires less time than the method using a rectangular window due to the influence of memory access time. Note that the proposed method demands less time and memory than the CIIR employing a triangular window, which achieves similar image quality to that of the proposed method in the above optical experiment.

Therefore, the experimental results show that the proposed CIIR provides reconstructed images with better subjective image quality than the standard CIIR. It also provides reconstructed images with similar image quality to that of the window-based CIIR using a triangular window. In addition, from an objective evaluation perspective, the proposed CIIR method requires less processing time due to less memory usage compared to that of the window-based CIIRs.

## 5. Conclusions

We have introduced a computational integral imaging reconstruction model via elemental image blending. Our signal model defines elemental image blending in detail from the perspective of the window. Unlike other methods, the proposed model does not require a normalization process. Moreover, our model is expected to provide enhanced image quality due to its continuous and smooth window-like behavior. Optical and computational experiments demonstrate that the proposed method improves image quality without normalization, also requiring less memory and processessing time.

## Data Availability

Data sharing is not applicable.
